# Genomic Organization and Differential Signature of Positive Selection in the Alpha and Beta Globin Gene Clusters in Two Cetacean Species

**DOI:** 10.1093/gbe/evt176

**Published:** 2013-11-20

**Authors:** Mariana F. Nery, José Ignacio Arroyo, Juan C. Opazo

**Affiliations:** ^1^Instituto de Ciencias Ambientales y Evolutivas, Facultad de Ciencias, Universidad Austral de Chile, Valdivia, Chile; ^2^Programa de Doctorado en Ciencias mención Ecología y Evolución, Facultad de Ciencias, Universidad Austral de Chile, Valdivia, Chile

**Keywords:** adaptive response, cetaceans, hemoglobin, gene family, positive selection

## Abstract

The hemoglobin of jawed vertebrates is a heterotetramer protein that contains two α- and two β-chains, which are encoded by members of α- and β-globin gene families. Given the hemoglobin role in mediating an adaptive response to chronic hypoxia, it is likely that this molecule may have experienced a selective pressure during the evolution of cetaceans, which have to deal with hypoxia tolerance during prolonged diving. This selective pressure could have generated a complex history of gene turnover in these clusters and/or changes in protein structure themselves. Accordingly, we aimed to characterize the genomic organization of α- and β-globin gene clusters in two cetacean species and to detect a possible role of positive selection on them using a phylogenetic framework. Maximum likelihood and Bayesian phylogeny reconstructions revealed that both cetacean species had retained a similar complement of putatively functional genes. For the α-globin gene cluster, the killer whale presents a complement of genes composed of HBZ, HBK, and two functional copies of HBA and HBQ genes, whereas the dolphin possesses HBZ, HBK, HBA and HBQ genes, and one HBA pseudogene. For the β-globin gene cluster, both species retained a complement of four genes, two early expressed genes—HBE and HBH—and two adult expressed genes—HBD and HBB. Our natural selection analysis detected two positively selected sites in the HBB gene (56 and 62) and four in HBA (15, 21, 49, 120). Interestingly, only the genes that are expressed during the adulthood showed the signature of positive selection.

## Introduction

Hemoglobin (Hb) is one of the best studied of all macromolecular proteins. The main function of Hb is to transport molecular O_2_ from the respiratory systems to the tissues, where it is released for cell use (Dickerson and Geis 1983). The Hb of jawed vertebrates is a heterotetramer protein that contains two α- and two β-chains, which are encoded by members of the corresponding α- and β-globin gene families. These families are ontogenetically regulated, which means that functionally distinct Hb isoforms are differentially expressed in embryonic and adult erythroid cells. They are also biochemically optimized for oxygen transport under different physiological conditions faced during all stages of development (Hardison 1998; Storz et al. 2008; Hoffmann et al. 2010). During the course of vertebrate evolution, the α- and β-globin gene families have undergone a complex history of gene duplication and divergence that have given rise to a great diversity in extant species (Hoffmann et al. 2008, 2010; Opazo et al. 2008a,b). These regulatory and functional divergences constitute an important source of variation that affects physiologically important aspects of hemoglobin properties (Hoffmann et al. 2008; Storz et al. 2008). From a genomic standpoint, α- and β-globin gene families are located in two different clusters in the genomes of amniotes.

Physiological adaptations to hypoxia are necessary in environments where oxygen availability is reduced. For example, evidence from a number of high-altitude vertebrates indicates that modification of Hb properties typically plays a key role in mediating an adaptive response to chronic hypoxia: high-altitude species generally have Hbs with higher O_2_ binding affinities than those of their lowland relatives (e.g., Weber et al. 2002; Storz 2007). In aquatic environment, diving vertebrates also have developed suitable mechanisms for the maintenance of an adequate O_2_ supply to tissues under hypoxic conditions (Tellone et al. 2000). For example, Hbs from emperor penguin (Meir and Ponganis 2009) and sea turtle (Petruzzelli et al. 1996) display a Bohr effect that appears well adapted for gas exchange during prolonged dives. It is likely that the Hb of diving marine mammals, which usually show a remarkable hypoxia tolerance, would possess adaptive changes as well. In this respect, cetaceans may have developed particular molecular mechanisms for the maintenance of adequate oxygen supply to tissues during acute hypoxia (Brix et al. 1990).

Although a few cetacean α- and β-globins have been sequenced so far and analyzed functionally (Brix et al. 1990; Tellone et al. 2000; Corda et al. 2003; Remington et al. 2007; Manconi et al. 2009), no previous work has characterized the α- and β-globins clusters on this particular lineage. Most of the earlier studies have shown that the oxygen-binding behavior of cetaceans’ hemoglobin has unusual properties probably linked to their peculiar diving behavior: lower intrinsic oxygen affinities and greater Bohr effects. These characteristics could enhance oxygen release to respiring tissues when exercise causes a drop in levels of ATP. Remington et al. (2007) highlighted that the relative low-oxygen affinities are intrinsic to the Hb’s structure and showed that genetic diversity was clearly responsible for the variations in Hb type and function of these animals.

In this context, it is tempting to think that Hb may have experienced a greater selection pressure to modify its functional properties, resulting in a molecule more adapted to prolonged diving behavior. Thus, α- and β-globin gene families are great candidates to determine whether the unusual properties of cetacean’s hemoglobin are attributable to differences in Hb gene repertoire and/or to positive selection in amino acid residues that play important roles in controlling Hb-O_2_ affinity (e.g., Perutz 1983; Berenbrink 2006; Storz and Moriyama 2008; Mandic et al. 2009). Accordingly, the objectives of this study were as follows: 1) to characterize the organization of the Hb gene clusters in cetaceans, 2) to infer orthologous relationships among duplicated copies of α- and β-globin genes, and 3) to infer the potential role of positive selection on the α- and β-globin genes in the cetacean lineage using a phylogenetic framework.

## Materials and Methods

### Sequence Data

We used bioinformatic tools to identify the full complement of structural genes in the α- and β-globin gene families in 13 laurasiatherian species and one primate as outgroup species (supplementary table S1, Supplementary Material online). We included four cetartiodactyls (dolphin, *Tursiops truncatus*; killer whale, *Orcinus orca*; cow, *Bos Taurus*; pig, *Sus scrofa*); two bats (megabat, *Pteropus vampyrus*; microbat, *Myotis lucifugus*); four carnivores (cat, *Felis catus*; dog, *Canis familiaris*; panda, *Ailuropoda melanoleuca*; ferret, *Mustela putorius*); one perissodactyl (horse, *Equus caballus*); and two insectivores (hedgehog, *Erinaceus europaeus*; shrew *Sorex araneus*). Genes were manually annotated by comparing known exon sequences to genomic contigs using the program Blast2seq, version 2.2 (Tatusova and Madden 1999), and also using the program Genscan (Burge and Karlin 1997). All sequences were aligned using the program MUSCLE (Multiple Sequence Comparison by Log-Expectation) (Edgar 2004). Putatively functional genes were characterized by an intact open reading frame with the canonical three exon/two intron structure typical of vertebrate globin genes, whereas pseudogenes were identifiable because of their high sequence similarity to functional orthologs and the presence of inactivating mutations and/or the lack of exons.

### Inferring Orthologous Relationships

We inferred orthologous relationships by reconstructing phylogenetic relationships among the α- and β-globin genes using Bayesian and maximum likelihood approaches, as implemented in Mr.Bayes v.3.1.2 (Ronquist and Huelsenbeck 2003) and Treefinder version October 2008 (Jobb et al. 2004). The models of nucleotide substitution were selected through the “propose model” tool of Treefinder version October 2008 (Jobb et al. 2004). For the Bayesian analyses, two simultaneous independent runs were performed for 30,000,000 iterations of a Markov chain Monte Carlo algorithm, with six simultaneous chains, sampling every 1,000 generations. Support for the nodes and parameter estimates were derived from a majority rule consensus of the last 15,000 trees sampled after convergence. In maximum likelihood, we estimated the best tree using the models of nucleotide substitution previously selected, and support for the nodes was estimated with 1,000 bootstrap pseudoreplicates. Because of the occurrence of interparalog gene conversion among globin genes (Storz et al. 2007; Opazo et al. 2008b), we used phylogeny reconstructions of noncoding sequences (intron 2 and flanking sequences, 1 kb upstream of the start codon, and 1 kb downstream immediately after the stop codon) to infer orthologous relationships among α- and β-globin genes.

### Detecting Positive Selection

To investigate the possible role of natural selection in the evolution of α- and β-globin genes in the cetacean lineage, we used the branch-site model as implemented in the program codeml included in the software package PAML v4.4 (Yang 2007). Because the branch-site analysis estimates rates of evolution on a codon-by-codon basis on a specific branch of the tree, its implementation is particularly useful in cases when different gene segments evolve at different rates. This model assumes that branches on the tree are divided into foreground branches, where some sites may be under positive selection, and background branches where positive selection is absent (Yang and Nielsen 2002; Yang et al. 2005; Zhang et al. 2005). Under this methodology, sites are categorized into four classes 0, 1, 2a, and 2b with proportions of *p*0, *p*1, *p*2a, and *p*2b, respectively. Site class 0 includes codons that evolve under purifying selection on both the foreground and background branches, with 0 < ω_0_ < 1. In site class 1, codons evolve neutrally in all lineages, with ω_1_ = 1, whereas in classes 2a and 2b, positive selection is allowed on the foreground branches with ω_2_ > 1, but not on the background branches. This model is compared with the corresponding null hypothesis of neutral evolution, where ω_2_ is fixed to 1. If the null hypothesis is rejected by the likelihood ratio test (LRT), a Bayes empirical Bayes approach is used to calculate the posterior probabilities that each site has evolved under positive selection on the foreground lineage (Yang et al. 2005). In all cases, three starting ω values (0.5, 1, and 2) were used to check the existence of multiple local optima. In our case, the ancestral branch of the cetacean clade was labeled as the foreground branch. All the analyses were based on a phylogeny that includes representative species of laurasiatherian mammals. The tree topology used to conduct the analyses of variable ω among lineages and sites is based on published literature (Nery et al. 2012).

## Results

### Genomic Structure of the Dolphin and Killer Whale α- and β-Globin Gene Clusters

**Fig. 1.—:**
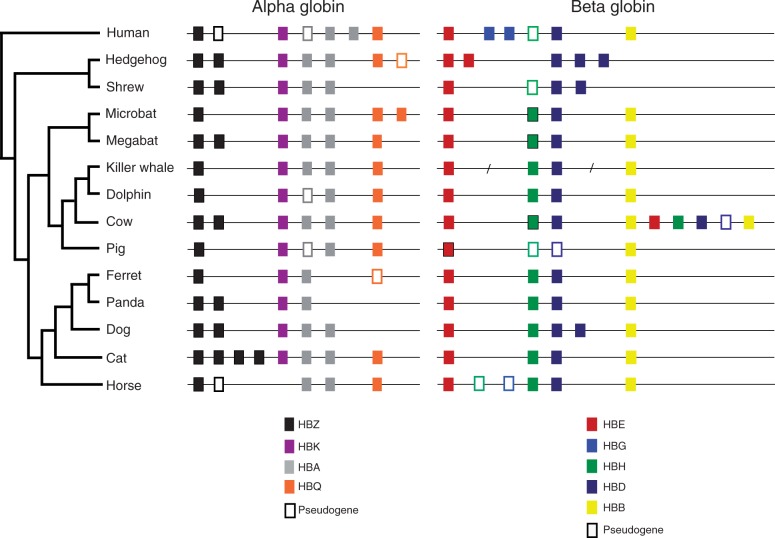
Genomic structure of α- and β-globin gene clusters in laurasiatherian species. The orientation of the cluster is from 5′ (on the left) to 3′ (on the right). Diagonal slashed on the beta-globin cluster of the killer whale indicates gaps in genomic coverage. Because of these gaps, the exon 3 of the HBD gene of the killer whale is missing.

**Fig. 2.—:**
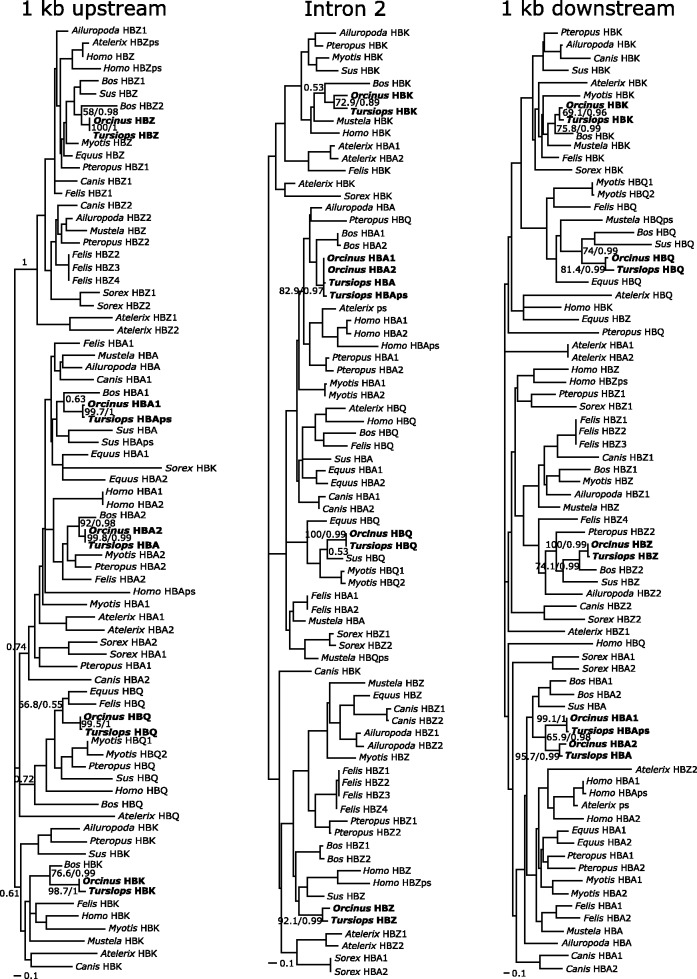
Maximum likelihood trees showing relationships among α-globin genes based on 1 kb of 5′ flanking sequences (left), intron 2 (center), and 1 kb of 3′ flanking sequence (right). Values on relevant nodes denote bootstrap support values and Bayesian posterior probabilities.

The α-globin gene cluster of the cetacean species included in our study appeared to have retained a similar complement of putatively functional genes: the killer whale presents a complement of genes composed of HBZ, HBK, and two functional copies of HBA and HBQ globin genes, whereas the dolphin possesses HBZ, HBK, HBA and HBQ genes, and one HBA pseudogene (fig. 1). Phylogenies based on noncoding sequences indicate that the α-globin genes found in the dolphin and in the killer whale are 1:1 orthologs to the α-globin gene repertoire present in other mammals (fig. 2). The α-globin gene cluster of the cetaceans retained very similar patterns of intergenic spacing in comparison to other laurasiatherian species: from the initiation codon of HBZ to the termination codon of HBQ, the gene cluster covered 11,690 bp in the dolphin, 10,601 in the killer whale, and 12,159 bp in the pig. Among laurasiatherian mammals, the number of putatively functional genes ranged from three in the ferret to eight in the cat. All species included in this study possess at least one functional copy of HBZ and HBA (fig. 1).

**Fig. 3.—:**
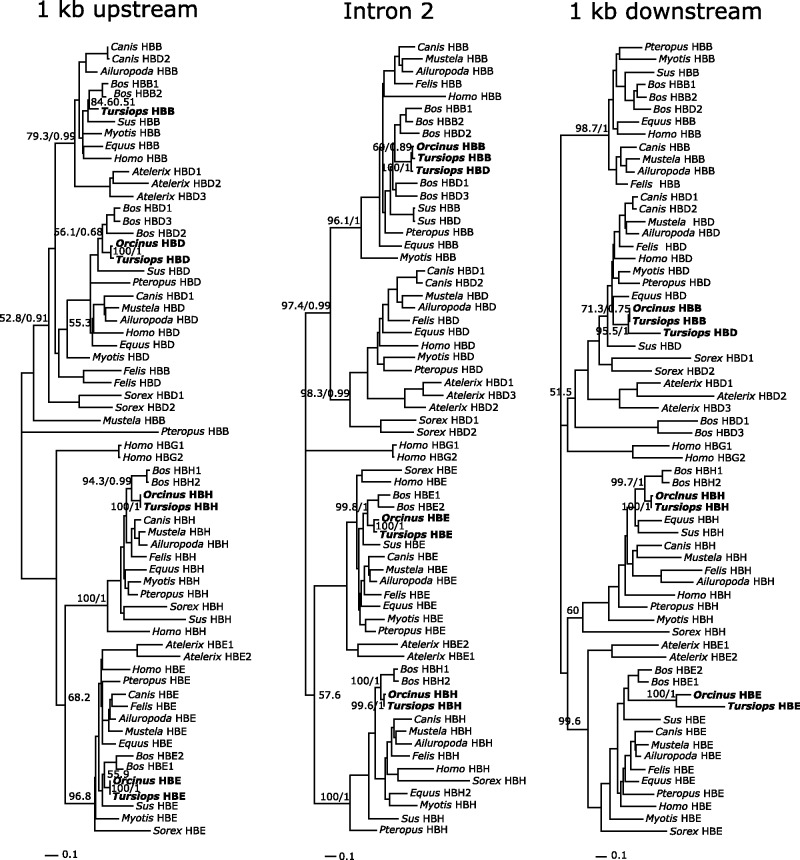
Maximum likelihood trees showing relationships among β-globin genes based on 1 kb of 5′ flanking sequences (left), intron 2 (center), and 1 kb of 3′ flanking sequence (right). Values on relevant nodes denote bootstrap support values and Bayesian posterior probabilities. The absence of killer whale HBB on the “1 kb upstream” tree is due to the poor quality of its genomic sequence.

For the β-globin gene cluster, cetaceans retained a complement of four putatively functional genes (from 5′ to 3′), two early expressed genes—HBE and HBH—and two adult-expressed genes—HBD and HBB (fig. 1). Phylogenies based on noncoding sequences indicate that the β-globin genes found in both the dolphin and the killer whale are 1:1 orthologs to the β-globin gene repertoire in other mammals (fig. 3). Also the dolphin β-globin gene cluster has remained similar in terms of intergenic spacing in comparison to other phylogenetically related species. From the first codon of HBE to the termination codon of HBB, the dolphin cluster spans 20,317 bp and the pig 19,516 bp. Unfortunately, the current state of the killer whale genome assembly does not permit inferences regarding patterns of intergenic spacing in the β-globin gene cluster. Among laurasiatherians, gene repertoire of the β-globin gene cluster varies from two functional genes in the pig up to eight in the cow, whose globins were involved in en bloc duplications (Townes et al. 1984).

### Variation in Omega Ratio

The results of the branch-site analyses are summarized in table 1. It is interesting to highlight that for both adult-expressed genes (HBA and HBB) the LRT indicates that the models which estimate a class of sites with an ω value higher than 1 had a significantly better fit than the null model in which the ω value was fixed to 1 [2Δℓ = 2*(ℓ_1_ − ℓ_0_) = 11.11 and 9.49; *p* = 0.008 and 0.002, respectively]. In these analyses, we were able to identify sites under positive selection in both genes. In the case of the HBA gene, sites 15, 21, 49, and 120 were positively selected, whereas for the HBB, gene sites 56 and 62 in HBB were inferred under the action of positive selection. In the case of the embryonically expressed genes (HBZ and HBE), the model A did not fit the data better than the null model in which the ω value was fixed to 1 [2Δℓ = 2*(ℓ_1_ − ℓ_0_) = 0 and 0.08; *p* = 1.0 and 0.69, respectively].

**Table 1:** Log Likelihood Values and Parameters Estimates under Different Branch-Site Models, Where the Cetacean Lineage Was Labeled as Foreground Branch

Gene	ln *L*	Model	Estimated parameters	Positively Selected Sites
HBZ (embryonic)	−2490.855	Null	*p*_0_ = 0.760, *p*_1_ = 0.238 *p*_2a_ = 0, *p*_2b_ = 0.001; ω_0_ = 0.001, ω_2a_ = 1.000, ω_2b_ = 1.000	NA
	−2490.855	Model A	*p*_0_ = 0.761, *p*_1_ = 0.239, *p*_2a_ = 0, *p*_2b_ = 0; ω_0_ = 0.064, ω_2a_ = 1.000, ω_2b_ = 1.000	NA

HBA (adult)	−2327.998	Null	*p*_0_ = 0.778, *p*_1_ = 0.191, *p*_2a_ = 0.024, *p*_2b_ = 0.006; ω_0_ = 0.050, ω_1_ = 1.000, ω_2a_ = 1.000, ω_2b_ = 1.000	NA
	−2322.444	Model A	*p*_0_ = 0.778, *p*_1_ = 0.184, *p*_2a_ = 0.031, *p*_2b_ = 0.007; ω_0_ = 0.050, ω_1_ = 1.000, ω_2a_ = 28.565, ω_2b_ = 28.565	15, 21, 49, 120

HBE (embryonic)	−2003.994	Null	*p*_0_ = 0.797, *p*_1_ = 0.166, *p*_2a_ = 0.031, *p*_2b_ = 0.006; ω_0_ = 0.049, ω_1_ = 1.000, ω_2a_ = 1.000, ω_2b_ = 1.000	NA
	−2003.917	Model A	*p*_0_ = 0.823, *p*_1_ = 0.171, *p*_2a_ = 0.005, *p*_2b_ = 0.001; ω_0_ = 0.049, ω_1_ = 1.000, ω_2a_ = 19.443, ω_2b_ = 19.443	17

HBB (adult)	−2296.787	Null	*p*_0_ = 0.651, *p*_1_ = 0.263, *p*_2a_ = 0.060, *p*_2b_ = 0.024; ω_0_ = 0.053, ω_1_ = 1.000, ω_2a_ = 1.000, ω_2b_ = 1.000	NA
	−2292.040	Model A	*p*_0_ = 0.702, *p*_1_ = 0.278, *p*_2a_ = 0.014, *p*_2b_ = 0.005; ω_0_ = 0.057, ω_1_ = 1.000, ω_2a_ = 24.033, ω_2b_ = 24.033	56, **62**

Note.—ln *L* likelihood value, *p* proportion of site class, ω omega value for site classes. Sites inferred under positive selection for HBA gene had posterior probabilities values of 0.60, 0.88, 0.56, and 0.55, respectively. For HBB gene, posterior probabilities values were 0.79 and 0.99, respectively.

## Discussion

The transition from land to an aquatic environment in the early evolutionary history of cetaceans involved extensive modifications on their physiological, anatomical, and behavioral systems (Butler 2004; Ramirez et al. 2007). One of the most challenging aspects of a breath-holding animal living in an aquatic environment is to deal with extended periods of limited oxygen during submersion. Cetaceans have developed several strategies to cope with this limitation, such as O_2_ saving by reduction of the metabolic rate, selective vasoconstriction to assure O_2_ supply in sensitive organs, massive overexpression of myoglobin in aerobic muscles, a modified hemoglobin that performs better under acute hypoxia conditions, among others (Brix et al. 1990; Ramirez et al. 2007). Given that modifications of Hb function are often implicated in adaptation to acute hypoxia and because much is known about the structure–function relationships of vertebrates Hbs and their role in blood–O_2_ transport, the study of Hb function in vertebrate species that have developed great hypoxia tolerance, such as cetaceans, provides a great opportunity to elucidate detailed molecular mechanisms of physiological adaptation.

### Cluster Organization in the Cetacean Lineage

The laurasiatherian globin gene repertoire typically comprises four functional genes for the α- (HBZ, HBK, HBA, HBQ) and β-globin gene clusters (HBE, HBG, HBB, HBD). In this regard, the cetacean species included in our study, in contrast to other lineages that underwent a complex history of gene turnover, present a typical gene repertoire for a laurasiatherian species (fig. 1). Not surprisingly, for the dolphin and the killer whale α- and β-globin clusters, the embryonic globin genes were located at 5′ of those with an adult expression, as is the general pattern in the globin gene clusters of vertebrates.

It has been suggested that the variation in the globin gene repertoire among different lineages may constitute an important source of variation that affects physiologically important aspects of blood oxygen transport (Weber 2007; Storz and Moriyama 2008; Storz et al. 2008). As diving mammals, which have to face prolonged periods of hypoxia during diving, one could expect that the globin genes in the cetacean lineage would show a complex history of gene turnover. But instead they appear to have retained a typical gene repertoire as other laurasiatherian mammals (fig. 1). Given this pattern, it is likely that the phenotypic differences are most attributable to changes in protein structure rather than in copy number variation and genomic organization.

### Variation in Selective Pressure

The α- and β-globin gene clusters are ontogenetically regulated and biochemically optimized for oxygen transport under the different physiological conditions that are encountered during the embryonic and adult stages of development. It is well known that the fetal hemoglobin is structurally different from the adult hemoglobin by possessing greater affinity for oxygen than the adult hemoglobin (Delivoria-Papadopoulos and McGowan 1998). As a consequence, fetal hemoglobin combines more rapidly with oxygen at low tension than does adult hemoglobin. This is what is needed because the partial pressure of oxygen in the arterial blood is considerably lower than that of the atmospheric environment at the sea level. It is then expected that both the embryonic and the adult globins might be subject to different selective regimes. Our selective pressure analyses reflected exactly this scenario: only the cetacean adult α- (HBA) and β-globin (HBB) presented signatures of positive selection, whereas the embryonic α- (HBZ) and β-globins (HBE) seem to be more constrained (table 1). This evolutionary pattern (i.e., embryonic genes evolving more slowly than those expressed in adults) is common in the literature, and it seems to be widespread on vertebrate genome (Goodman 1963; Shapiro 1991; Roux and Robinson-Rechavi 2008). There are two nonmutually exclusive hypotheses to explain this pattern. The “developmental constraint” hypothesis suggests that genes expressed in early developmental processes are under strong negative selection as a means to avoid mutations that could cause deleterious cascading effects (Raff 1996). The “selection opportunity” hypothesis suggests that later stages in ontogeny give rise to greater opportunity for selection to act due to the exposure to varying environments (Darwin 1872; Gould 1977). Our results support both predictions of these models (embryonic globins under negative selection and adult globins subject to greater positive selection pressure).

**Fig. 4.—:**
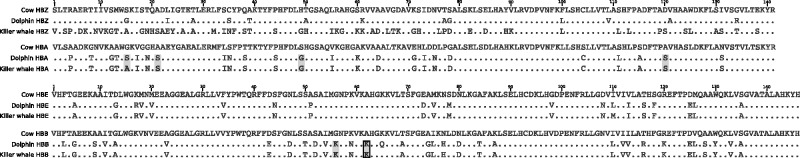
An alignment of HBA and HBB amino acid sequences from cow, dolphin, and killer whale. Positively selected amino acid sites are shaded.

The branch-site analyses identified four positively selected sites in the cetacean HBA gene—15 (Ser), 21 (Ser), 49 (Gly), and 120 (Ser)—and two sites in the cetacean HBB gene—56 (Lys) and 62 (Lys). Interestingly, almost all these substitutions are shared by both cetacean species, indicating that positive selection in the common ancestor of these species was responsible for the remodeling of this protein (fig. 4). It is known that Hb–oxygen affinity can be modified by amino acid substitutions that decrease or enhance the structure for oxygen binding or by changing the affinity of Hb for allosteric effectors (Perutz 1989). The residue 62 is a positively selected site in HBB gene and is known to be part of the E helix of the beta chain. According to the three-dimensional model of Perutz et al. (1968), some residues of the E helix seem to be essential in the maintenance of the heme in the nonpolar pockets of the alpha and beta chains.

Regarding the positively selected sites in the HBA gene, the residue 21 (Ser) is almost always occupied by an alanine or a valine on eukaryotes, and in few species by leucine or isoleucine (Harteveld et al. 2007). The presence of a serine at this position found in both cetacean species (fig. 4) was already described as naturally occurring in human but the physiological consequences for this specific mutation are still unknown (Harteveld et al. 2007). The site 120 (Ser) is known to be involved in the α1β1 contacts in the Hb molecule (Perutz and Lehmann 1968; Sacks et al. 1978), and consequently, an amino acid substitution at this site causes abnormality for oxygenation, as already described by studies that found new variants at this residue (Harano et al. 1989; Dinçol et al. 2006).

We did not obtain the oxygen dissociation curve from the dolphin Hb and the physiological effects of the positively selected residues 56 in HBB, and 15 and 49 in HBA are yet to be determined. However, other studies already described natural mutants occurring at these positions in human, with different amino acids (Szelényi et al. 1980; Turbpaiboon et al. 2002; Chang et al. 2002; Akar et al. 2003; Williams et al. 2007).

Although not identified as a positively selected site, we noted that the residue 65 of the HBB gene is occupied by a glutamine residue (Gln) only in the dolphin species (fig. 4). This residue is located very close to the distal histidine E7, an invariant amino acid site, which is implicated in the oxygenation process (Rohlfs et al. 1990). Garel et al. (1976) described a human hemoglobin variant in which a glutamine residue—the same found in the dolphin—substitutes the lysine residue in position 65. They found that this new variant results in a moderate decrease in cooperativity without changing the Hb stability. It suggests that hemoglobin with a 65Gln will result in a protein with decreased oxygen affinity. In diving birds and mammals, the Hb-O_2_ affinities are not high, because they do not experience environmental hypoxia while breathing (i.e., low O_2_ partial pressure; Snyder 1983; Willford et al. 1990; Ramirez et al. 2007). These animals may instead benefit from an Hb with lower intrinsic oxygen affinities, which would enhance Hb-O_2_ unloading during acute hypoxia, and also a high Bohr effect, which would intensify O_2_ unloading when acidosis increases during diving (Willford et al. 1990; Butler and Jones 1997; Ramirez et al. 2007).

It is important to note that inferences about functional divergence based on omega variation among lineages do not replace research on the biochemical properties of proteins. Nevertheless, our analyses is the first informative step toward understanding the functional divergence and produce candidates to perform site-directed mutagenesis studies and confirm whether or not these specific amino acid substitutions confer cetacean’s hemoglobin with unique properties that underlie their adaptation to the acute hypoxia.

### General Conclusions

Taken together, our analyses represent a step toward increasing our knowledge about the α- and β-globin genes in the cetacean lineage and how they evolved. Our study revealed that α- and β-globin gene families of the dolphin and killer whale do not present a complex history of duplication and divergence; instead, they appeared to have retained a typical laurasiatherian gene repertoire. Our branch-site analyses were able to identify positively selected sites only across the adult α- and β-globin genes, reinforcing the interesting pattern already described for other species, where embryonic genes appear to evolve slower than the adult expression genes (Goodman 1963).

## Supplementary Material

Supplementary table S1 is available at *Genome Biology and Evolution* online (http://www.gbe.oxfordjournals.org/).

Supplementary Data
