# Supplementary material for: Genomic Organization and Differential Signature of Positive Selection in the Alpha and Beta Globin Gene Clusters in Two Cetacean Species
Source: Genome Biol Evol. 2013 Nov 20;5(12):2359–67. doi: 10.1093/gbe/evt176 (PMC3879965; doi:10.1093/gbe/evt176)
Supplement: Supplementary Data [file supp_5_12_2359__index.html]

Genomic organization and differential signature of positive selection in the alpha and beta globin gene clusters in two cetacean species — Genomic Organization and Differential Signature of Positive Selection in the Alpha and Beta Globin Gene Clusters in Two Cetacean Species — Supplementary Data 

# Genomic Organization and Differential Signature of Positive Selection in the Alpha and Beta Globin Gene Clusters in Two Cetacean Species

## Supplementary Data

files

**Files in this Data Supplement:**

- Supplementary Data - docx file
