# Supplementary material for: Genomic Organization and Differential Signature of Positive Selection in the Alpha and Beta Globin Gene Clusters in Two Cetacean Species
Source: Genome Biol Evol. 2013 Nov 20;5(12):2359–67. doi: 10.1093/gbe/evt176 (PMC3879965; doi:10.1093/gbe/evt176)
Supplement: Supplementary Data [file supp_evt176_Supplementary_Table_1.docx]

**Supplementary Table 1.** Accession numbers of all sequences and taxa used in this study.

|  |  | **Alpha Globin** | **Beta Globin** |
| --- | --- | --- | --- |
| **Species** | **Common name** | **Accession No.** | **Accession No.** |
| *Myotis lucifugus* | Microbat | AC182000 | AC127473 |
| *Pteropus vampyrus* | Megabat | Scaffold 3459 | Scaffold 2552 |
| *Sorex araneus* | Shrew | AC166625 | AC166888 |
| *Erinaceus europeus* | Hedgehog | AC150435 | AC171157 |
| *Equus caballus* | Horse | AC203695 | AC209111 |
| *Bos taurus* | Cow | AC130788 | AC151111 |
| *Sus scrofa* | Pig | AC130791 | AC126924 |
| *Canis familiaris* | Dog | AC183635 | AC127473 |
| *Felis catus* | Cat | AC130194 | AC129072 |
| *Tursiops truncatus* | Dolphin | Scaffold 2227 | Scaffold 93543 |
| *Orcinus orca* | Killer whale | ANOL02025688.1 | ANOL02052691, ANOL02052692, ANOL02052693 |
| *Alluropoda melanoleuca* | Panda | 193383 | Scaffold 13682 |
| *Mustela putorius* | Ferret | GL896925.1 | GL897188.1 |
| *Homo sapiens* | Human | NG000006 | AC104389 |
